# Microbial consortia for the conversion of biomass into fuels and chemicals

**DOI:** 10.1038/s41467-025-61957-x

**Published:** 2025-07-21

**Authors:** Derek T. Troiano, Michael H.-P. Studer

**Affiliations:** School of Agricultural, Forest, and Food Sciences, Bern University of Applied Sciences, Zollikofen, Switzerland

**Keywords:** Metabolic engineering, Applied microbiology, Microbiology techniques, Microbiome

## Abstract

There is currently significant interest in employing microbial communities for converting lignocellulosic feedstock into chemicals, fuels, and other products of use to humans. Both naturally occurring microbial communities, which can be prohibitively complex, and synthetic consortia, which are simple though can be unstable and unpredictable, have been employed to that end. Recent work has focused on developing tools for enabling wider application of microbial consortia in both lignocellulose valorization and bioprocesses in general. Together with improved methods of process monitoring and creative process design, newly developed biosynthetic tools may represent key facilitators for commercial realization of consortia-based lignocellulose conversion processes.

## Abstract (web summary)

Microbial consortia have attracted much recent interest in the conversion of lignocellulosic feedstocks into valuable chemicals. Here, the authors provide an overview of recent developments, identify limitations to commercialization, and summarize available strategies and tools for addressing such limitations.

## Introduction

**Fig. 1:**
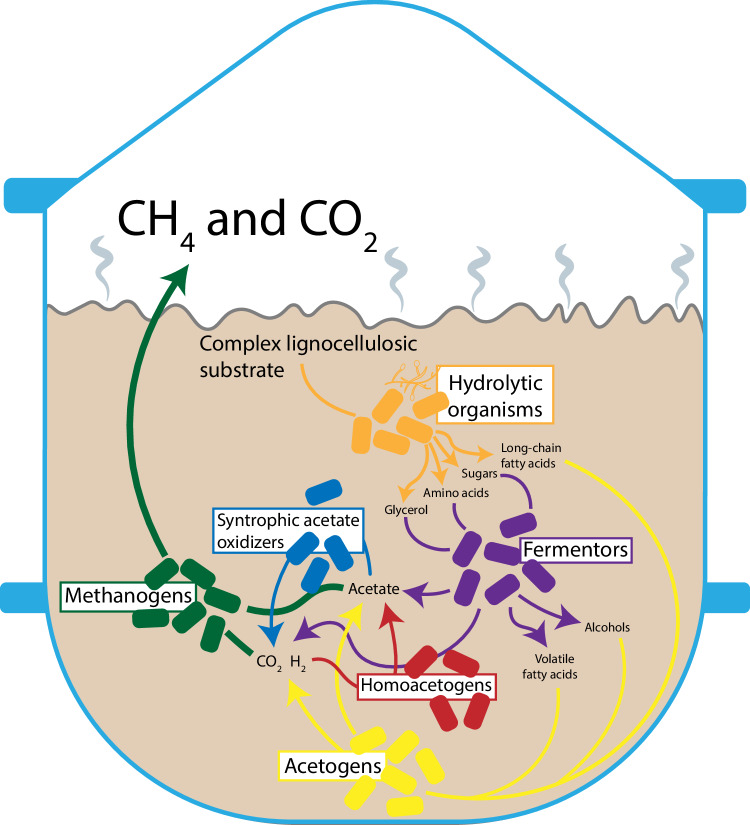
Anaerobic digestion: a representative example of a diverse microbial community with complex interactions. A simplified overview of anaerobic lignocellulosic substrate conversion begins with a hydrolysis phase wherein hydrolytic fungi and bacteria break down the cellulose and hemicellulose into soluble organic molecules. Most of these products are converted in an acidogenesis step by fermenting microbes into alcohols, organic acids (including acetate), hydrogen and CO_2_. In acetogenesis, the long chain fatty acids (from the hydrolysis step), volatile fatty acids, and alcohols are converted by acetogenic microbes into acetate, hydrogen, and CO_2_. Finally, in methanogenesis, methanogenic archaea convert the acetate (acetoclastic) or the CO_2_ and hydrogen (hydrogenotrophic) into methane and CO_2_. Also notable within the community are syntrophic acetate oxidizing and homoacetogenic microbes which respectively convert acetate to CO_2_ and hydrogen and the reverse reaction^7,98,99^.

As part of the multiform effort to obviate fossil-based resource consumption, biomass represents a viable source of carbon feedstock for use in the production of materials and chemicals. While theoretically carbon neutral, biomass can present ecological, economic, and/or ethical issues depending on the specific source^1,2^. Many of these issues may be avoided by focusing efforts specifically on lignocellulosic biomass which is inedible, generally cheap, and, if using waste residues, does not require additional land or resources for production^2^. Still, lignocellulosics present unique technical challenges related to a complex and recalcitrant composition which hampers conversion of these materials into products with commercial/industrial value^2,3^. Beyond pulp and paper, lignocellulosic-based processes have only been implemented to a limited extent on a commercial scale. For example, while ethanol is the most important bio-based chemical in terms of production volume, the largest producer of ethanol, the United States (~55 billion liters in 2022), produces an estimated < 0.01% using lignocellulosic feedstock^4,5^. Other products including organic acids, biopolymers (e.g., polylactic acid), and other biofuels (e.g., butanol) may also be derived from lignocellulose and present promise as viable alternatives to respective fossil fuel-based analogues but remain largely in early, pre-commercial stages of development. The main challenge for most, if not all, of these products is the high cost of production from lignocellulose^3^. Notably, one by now commercially well-established product of lignocellulosic feedstocks is biogas, which is produced from agricultural residues, manure, green waste, etc. Integral to biogas production processes is the exploitation of anaerobic microbial communities with diverse and complex interactions as outlined in Fig. 1^6^.

**Fig. 2:**
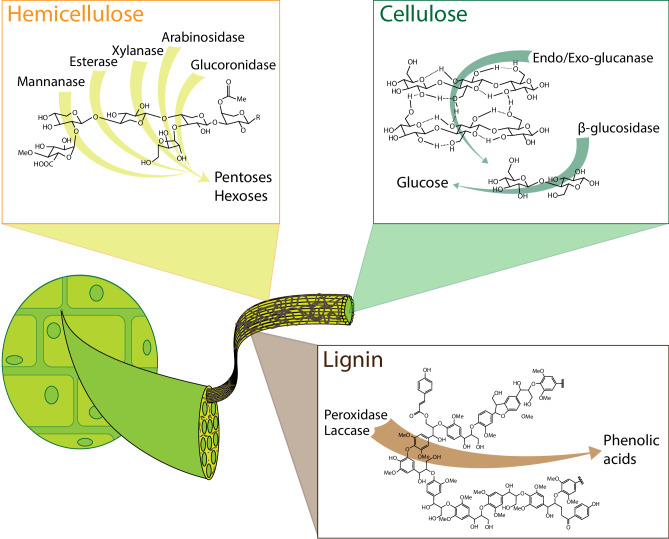
The biopolymers of lignocellulosic plant cells. Lignocellulosic plant cell walls mainly comprise three interconnected biopolymers: (i) cellulose, a linear homopolymer of β−1,4-glycosidic bond-linked glucose molecules in repeating units of cellobiose which form crystalline microfibrils via hydrogen bonding; (ii) hemicellulose, an amorphous, branched heteropolymer comprising various pentose sugars and sugar acids which forms a matrix around cellulose; and (iii) lignin, an aromatic heteropolymer which fills the space between hemicellulose and cellulose fibers (structure shown here adapted from Bertella and Luterbacher^100^). The compositional diversity of lignocellulosic fibers requires many different types of specialized enzymes -and in turn diverse organisms which specialize in their production- for complete degradation.

The industrial conversion of lignocellulose into biogas by microbial communities reflects what is observed in nature. Whether it be in the soil, water, gut of a ruminant, etc., the carbon and nitrogen within lignocellulosic biomass is transformed via the cooperative action of diverse communities of microbes^6,7^. These communities function in such a way that different members of the community specialize in different sub-functions to synergistically perform complete lignocellulose degradation (e.g., the various biopolymers which comprise biomass are degraded by specific enzymes produced by different microbes as illustrated in Fig. 2)^8,9^. Rather than a single microbial strain evolving to perform the numerous tasks required for catabolizing lignocellulose, division of labor evolved among these communities as a more energetically efficient strategy (i.e., metabolic burden on the individual is lower)^6–9^. Moreover, these co-evolved communities exhibit myriad interactions which make them eminently stable and robust (e.g., resistance to system perturbations such as environmental changes, contamination, and toxins)^7,9^. Such natural phenomena may therefore serve to inform solutions to current challenges facing wider industrial exploitation of lignocellulosic feedstocks including efficient conversion of all components of lignocellulosic biomass into products (i.e., not just the cellulose-derived glucose) and reduction of individual processing steps via consolidation.

While there are indeed increasingly numerous reports in the scientific literature of microbial consortia being employed in the conversion of diverse lignocellulosic feedstocks into a variety of valuable chemicals, there is despite this, little evidence of increased commercial application. The aim of this review is therefore to first provide an overview of recent notable reports on microbial consortia-based lignocellulose conversion processes and then to identify both current limitations to commercialization as well as key developments in strategies and tools applicable for addressing such limitations. Use of microbial consortia is already seen as a key theme in the future of biochemical engineering as a whole^10^ and thus the field of biomass conversion may benefit from concurrent progress being made across bioprocess disciplines.

## Microbial consortia can enable integrated lignocellulose conversion

Lignocellulosic biomass is primarily comprised of three biopolymers, cellulose, hemicellulose, and lignin, which are themselves primarily comprised of hexose sugars, pentose sugars, and phenolic compounds, respectively. Typically, unprocessed biomass is first pretreated (e.g., using steam which facilitates solubilization of the biopolymers including the lignin fraction^11–13^) to render the biopolymers amenable to subsequent hydrolysis. Currently, most of the carbon embedded in bio-products (in terms of volume, predominantly represented by ethanol) is derived solely from glucose/starch and thus the most mature bioconversion technologies are largely not based on lignocellulosic substrates^4,5,14,15^. Of those that are, traditionally the reliance has similarly been on the hexose content in lignocellulose (i.e., glucose from cellulose and, to a lesser extent, mannose from hemicellulose) as this is readily fermentable by typical industrial microbes (e.g., *S. cerevisiae*)^15^. Most of the hexose in lignocellulose is in the cellulose fraction which must be subjected to a cocktail of enzymes generally including exocellulases, endocellulases, and β-glucosidases that act on the polymer reducing end, internal bonds, and cellobiose (the product of exo- and endo- cellulases) respectively to release the glucose^16^. In commercial cellulosic ethanol production, this glucose is then converted into ethanol via fermentation with yeast or, in some cases, *Zymomonas*^3^. Though lignocellulosic biomass can be highly variable, hexose comprises on average less than half the dry mass of a given biomass residue. As feedstock costs can represent >30% of overall biorefinery operation costs^17^, one key strategy for improving the economics of biorefining processes is therefore to convert a larger fraction of the feedstock into product^15^. This would additionally improve the conformance of biorefining operations to green principles of chemical production (i.e., improved atom economy)^18^.

As pentoses in hemicellulose can represent ~35% of the mass in lignocellulose, much effort has been made to engineer industrial microbes capable of converting both hexose and pentose sugars (i.e., co-fermentation)^15^. Moreover, these organisms need to be tolerant, or engineered to be tolerant, of the inhibitors that are present in native as well as pretreated lignocellulosic biomass substrate, i.e., biomass hydrolysate^15^. While engineering a single strain to perform many non-native tasks can present advantages (e.g., a simple bioprocess), it also presents many disadvantages including high metabolic burden on the individual organism which can exhibit slow growth (e.g., due to intracellular resource competition) and/or loss of functionality over time (as now there is a selective advantage for mutants with loss of function)^19–21^. This energetic inefficiency is otherwise avoided by instead distributing tasks across multiple sub-populations of cells, whether through combinations of native specialists or through a variety of engineered strains^20^.

For example, co-cultures of glucose-, arabinose-, or xylose-fermenting yeast specialists have demonstrated both higher sugar conversion rates and better long-term functional stability than generalist yeast strains which lose pentose fermenting ability over time in favor of glucose specialization^22^. Functional stability is a key industrial advantage as it enables recycling of microbial biomass from cycle to cycle. This, in turn, obviates the need to produce a new inoculum with each cycle and enables long term evolution of the consortia which can lead to improved consortia characteristics such as, for example, inhibitor tolerance^22^. Worth noting here is the challenge related to separating lignin from microbial cells including yeast which hampers the recycling of microbial biomass in systems involving lignin-based feedstock^23–25^. One limitation observed in the yeast co-culture was the reduced growth rate of one of the xylose fermenting strains vis-à-vis growth in monoculture^22^. A possible cause of this was resource competition among the three different yeast strains. This underscores the need for improved understanding of microbial interactions within a consortium as well as strategies to address imbalances such as faster/slower growing strain combinations. To that end, spatial separation of imbalanced strains has been shown to effectively address this issue as in an example involving immobilization of glucose- and xylose- fermenting yeast strains in separate hydrogels which demonstrated added benefits of long-term reusability and storage^26^.

While all the carbohydrate content of lignocellulosic biomass is contained within cellulose and hemicellulose, the remaining ~20% of mass is lignin and all its aromatic building blocks (lignin represents the largest renewable source of aromatics^27^). Generally, lignin-derived feedstock streams comprise a mixture of compounds including vanillic acid, coumaric acid, and hydroxybenzoic acid. Thus, one promising approach for valorizing lignin is to funnel these various compounds through a microbial central metabolic pathway (usually through acetyl CoA) to produce one or one type of valuable product. Most reports of microbial biological funnels involve bacterial species such as *Pseudomonas* (specifically, *P. putida*) and *Rhodococcus* in the production mainly of biopolymers or polymer precursor molecules. For example, *P. putida* has been shown to convert lignin feedstock into cis,cis-muconic acid^28^ (which in this study was subsequently converted into adipic acid and then into 6,6-nylon) as well as itaconic acid^29^ and polyhydroxyalkanoates^30^. *Rhodococcus* has also shown use in production of cis,cis-muconic acid^31^ from lignin substrate in addition to pyradine carboxylic acids (which may serve as substitutes for terephthalic acid in polyester synthesis)^32^ and lipids^33^.

Though seemingly well-suited to the task of converting complex lignin substrate into products, there are few reports of microbial consortia employed for this purpose. Of the few, two and three strain *Rhodococcus* co-cultures have been described for conversion of lignin substrate into lipids and have presented advantages in, for example, conversion efficiency as compared with monocultures^34,35^. On the other hand, microbial consortia, including filamentous fungal consortia, have been applied in specifically degrading/depolymerizing lignin^36^. Recent advances in understanding the pathways involved in filamentous fungal catabolism of lignin demonstrate that filamentous fungi can uptake the carbon from lignin into central metabolism as opposed to external mineralization of lignin compounds to CO_2_^37^. Thus, future work may involve extending the role of filamentous fungi for inclusion in microbial consortia-conversion of lignin into valuable chemicals.

**Fig. 3:**
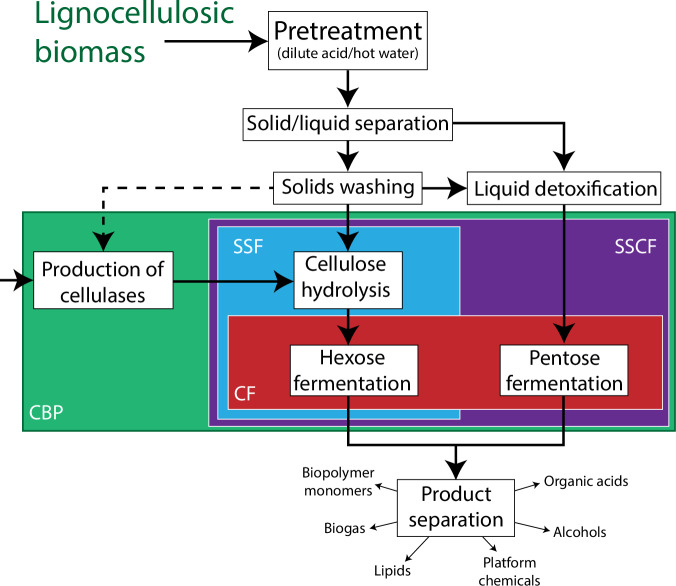
Typical lignocellulosic biomass conversion flow diagram. Lignocellulose must first be pre-treated following which the liquid and solid fractions are generally separated. Hydrolytic enzymes are used to convert the resulting biopolymers into the simple sugars that may then be converted into various products. Many of these individual unit operations have the potential to be combined into consolidated processes: combination of hexose and pentose fermentation into a single co-fermentation step (CF); combination of hydrolysis and fermentation into single step for simultaneous saccharification and fermentation (SSF); a combination of CF and SSF into simultaneous saccharification and co-fermentation (SSCF); and a combination of SSCF with in-situ production of hydrolytic enzymes in a single consolidated bioprocess (CBP). The terms used to describe these processes vary widely and it is worth noting that their employment here, particularly the use of CBP, is not universally standard.

In parallel with ensuring as much feedstock mass as possible is embedded into product, another key strategy in improving the economics of lignocellulose conversion is reducing individual processing steps (i.e., pre-treatment, hydrolysis, production) via consolidation thereby reducing costs associated with additional reactors, labor, time, etc^38^. (see Fig. 3). Just as in the above-described bioprocesses, the division of labor which characterizes microbial consortia make them perfectly suited for this task. Among the many iterations of this general strategy reported in literature, perhaps the most common involves consolidating the hydrolysis and fermentation steps. Some reports describe the combination of hydrolytic enzymes along with a fermenting microbe or microbes, as in one study which employs a co-culture of hexose- and pentose- fermenting yeasts (*S. cerevisiae* and *P. stipitis*, respectively) along with hydrolytic enzymes in order to convert food waste into ethanol^39^. Such an approach eliminates the need for a separate hydrolysis reaction but still requires the separate procurement of the enzymes used in hydrolysis.

In the interest of avoiding costs associated with cell-free enzymes, microbial whole-cells may be directly employed to produce hydrolytic enzymes in-situ. To that end, various reports describe monoculture engineering efforts which equip fermenters with cellulolytic capabilities (as in one study which engineered surface expressing hemicellulases on a xylose fermenting yeast to convert corn cob liquor to ethanol^40^), as well as the enhancement of chemical production in native lignocellulolytic organisms (e.g., the engineering of *Myceliophthera thermophila* to be capable of producing important bulk chemicals, 1,4-diacids, from either crystalline cellulose or plant biomass^41^). As with co-fermentation, a potentially more energetically efficient approach than engineering multiple non-native functions into a single organism may be to employ a consortium of microbes which distribute the individual tasks among population members. Natural communities of such organisms, which have coevolved for the specific purpose of converting lignocellulosic biomass, are available and indeed already employed in industrial settings including, as mentioned, in anaerobic fermentation for biogas production^6^. In addition to biogas, naturally-derived (and subsequently enriched) microbial consortia originating from, for example a wastewater treatment plant and the soil of a wheat field, have also been employed in the production of biohydrogen from corn stover^42^ and ethanol from wheat straw^43^, respectively. However, the challenge in using natural communities relates to their complexity^9^. Such communities can contain many hundreds of individual members making it untenable to characterize and understand all interactions thus prohibiting fine behavioral control and/or expansion of functionality^9^. Alternatively, discrete specialist organisms may be selected and combined into what is referred to as a synthetic consortium. These communities can be relatively simple and involve, for example, just a single discrete hydrolytic organism along with a discrete product specialist as in the reported combination of the hemicellulose-producing bacterium *Thermoanaerobacterium thermosaccharolyticum* with the succinic acid-producing specialist *Actinobacillus succinogenes* for the conversion of corn cob into succinic acid^44^. The filamentous fungus *Trichoderma reesei* is one of the most commonly employed cellulolytic specialists in lignocellulosic co-culture schemes due both to high enzyme producing capacity (it is the primary microbe employed in commercial cellulase production) and its ready cooperativity with a variety of other microbes in the conversion of cellulosic substrate including other filamentous fungi^45^, yeast^45^, bacteria^46,47^, or entire microbial consortia^48,49^.

The individual members of synthetic consortia need not only perform one task (i.e., either hydrolysis or product accumulation) as in several recent studies which rely on the hydrolytic organism to also contribute to product generation. In one such study, an oleaginous and lignocellulolytic fungus (*Aspergillus tubingensis*) was used to pretreat palm residues which were then, in a separate step, further converted via SSF involving *A. tubingensis* and the oleaginous yeast *Yarrowia lipolytica*^50^. When accounting for lipid accumulation across all steps and organisms, a yield of >150 mg/g lignocellulosic residue was obtained^50^. In this example, the fungus used for pre-treating the feedstock was evaluated in terms of its ability to reduce the lignin content of the feedstock, but it is unclear if the carbon from the lignin was ultimately used for lipid production. As mentioned, conversion of lignin into product is key to improving feasibility of lignocellulose conversion processes. In another study involving multiple producer strains, here employed in the conversion of alkali-extracted deshelled corn cobs into butanol, the cellulolytic bacterium (*Clostridium cellulovorans*) was engineered to also produce butanol and was combined with a primary butanol producing bacterium (*Clostridium beijerinckii*)^51^. Notable in this study was the incompatible pH requirements of the two strains. In an earlier study, this was overcome via sequential inoculation and separation of the bioprocess into multiple phases, while for the present study, *C. cellulovorans* was engineered to tolerate the low pH requirements of *C. beijerninckii* (in addition to butanol production capability) which obviated the need for sequential inoculation and ultimately improved productivity by >30%^51^.

Balancing the requirements and unique physiologies of individual consortium members is a key challenge in general for synthetic consortium-based bioprocesses, particularly as they become more complex and involve members from various domains and kingdoms of life. Desired combinations of microbes may have different environmental requirements (e.g., pH and oxygen) or possess significantly different growth rates presenting the risk of one member outcompeting another and driving it to extinction. Already mentioned for use in resolving different environmental requirements (i.e., pH), sequential inoculation may also be employed for resolving unbalanced growth rates as in one study involving a co-culture of *T. reesei* and the faster growing, itaconic acid-producing yeast *Ustilago maydis* where it was necessary to inoculate the substrate (here α-cellulose) with *T. reesei* at least several days before the yeast^52^. However, this approach is employed at the expense of productivity. Thus, other strategies for resolving conflicting microbial requirements have emerged including the engineering of spatial niches. For example, in order to accommodate incompatible oxygen requirements of aerobic cellulolytic fungi and anaerobic bacterial product specialists, membrane bioreactors with gas permeable membranes have been reported which cultivate spatially dependent aerobic/anaerobic niches^46,49^. In these systems, the aerobic fungus grows directly on the membrane where oxgen is abundant (the aerobic niche) and forms a biofilm across which the oxygen concentration decreases as a gradient. In one example of such a system, *T. reesei* was combined with the anaerobic, lactic acid-producing, and xylose-fermenting bacterium *Lactobacillus pentosus* to convert detoxified steam-pretreated beech wood into lactic acid^46^. The lignocellulosic substrate was hydrolyzed by the fungus into sugars which the *Lactobacillus* could then convert into lactic acid, a product which subsequently served as a platform for generation of a variety of other products via inoculation of additional lactic acid-fermenting bacteria^46^. Depending on the desired product, *Clostridium tyrobutyricum*, *Veillonella criceti*, or *Megasphaera elsdenii* were added to the reactor for the conversion of lactic acid into butyric acid, propionic and acetic acid, or higher short chain fatty acids (valeric and caproic acids)^46^. While sequential inoculation was employed, e.g., to allow accumulation of lactic acid prior to addition of the lactic acid converting bacteria, the approach ultimately enabled continuous production of fatty acids^46^. In a more recent study involving a similar pairing of strains (i.e., the cellulolytic fungus *Trichoderma asperellum* in combination with lactic acid producing *Lactobacillus paracasei*), niche formation was realized through hydrogel encapsulation of the anaerobic strain which in turn served as support for aerobic fungus biofilm formation^47^. Notably, microbes chosen in this study were first identified from decaying wood samples, thus demonstrating an approach which combines both use of natural consortia and, following identification of key members of this consortia, reconstruction into a synthetic consortia^47^.

The proliferation of reports in the scientific literature on microbial consortia employed in the conversion of lignocellulose into fuels and chemicals contrasts the absence of larger scale and/or commercial applications of such bioprocesses. Significant and interrelated bottlenecks in the advancement of consortia-based bioprocesses (in biorefining as well as applications in human health, environment, agriculture, etc.) are formed by the lack of adequate methods and tools for understanding, predicting, monitoring, controlling, and expanding functionality of these complex communities. Only microbial consortia that are predictable, stable, and tunable will be industrially relevant.

## Engineering stable and tunable synthetic microbial consortia bioprocesses

**Fig. 4:**
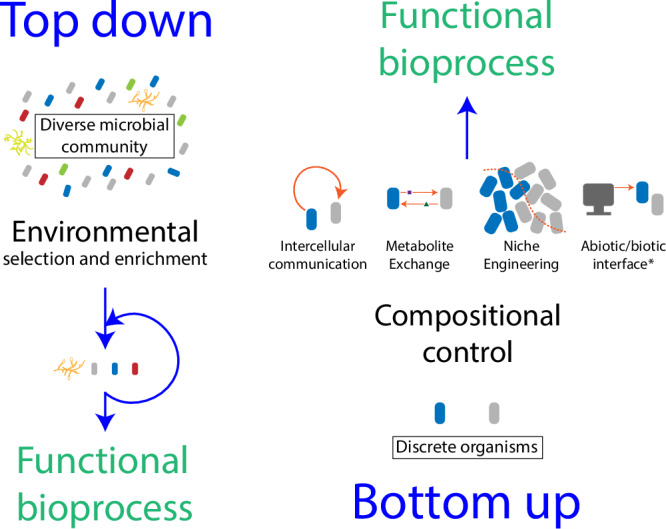
The two fundamental approaches to developing microbial consortia for use in bioprocesses. Top-down strategies typically involve complex microbial consortia isolated from natural systems that are modified to be simpler and/or more effective at a specific given task. This is achieved through environmental selective pressures which may remove undesirable consortia members while enriching the consortia with the desired microbes. A bottom-up approach involves rational combination of discrete organisms with known and desirable functionalities. Since these organisms often exhibit incompatible environmental requirements or physiological characteristics (i.e., different growth rates), strategies to ensure compatibility and stability are required for developing a functional bioprocess. The most widely reported strategies include the exploitation of intercellular communication networks (i.e., quorum sensing), metabolite exchange, niche engineering, and using abiotic input (*including light or electronic signals, i.e., optogenetics and electrogenetics) to modulate strain behavior.

Given the number of individual members within microbial consortia and the high complexity of metabolic interactions between even few members, characterization of consortium dynamics has been challenging and generally lacks comprehensiveness^7,9^. Consortia-based bioprocesses have therefore mostly been developed using one or a combination of two relatively ad-hoc approaches, by which all previously described examples may be characterized (see Fig. 4). A top-down approach involves exploitation of natural microbial communities which have already co-evolved to perform a function that happens to be of human interest. Though these communities may be modified (e.g., enriched with desirable microbes while selecting against microbes that may be detrimental to the desired function^7^, or through construction of niches which promote use of different metabolic pathways and enable tuning of product composition^49^), a comprehensive understanding of community dynamics is not necessarily required. While enriched communities are often employed for investigating key genes, pathways, and interactions, these communities may still contain prohibitively numerous members and, moreover, may lack key members (e.g., keystone organisms) for optimal functioning of the consortium^9^. Long considered in animal ecology and more recently applied to microbial communities, the identification, improved understanding, and retention of keystone organisms (i.e., organisms which individually or in a guild exert a considerable influence on microbiome structure and functioning irrespective of their abundance across space and time) is essential to successful exploitation of natural microbial communities^10,53^. As it is not always known in advance the roles of all members of the natural community, these organisms may be inadvertently selected against during the enrichment process, or it may not even be possible to culture them outside of their natural environment^9^. Lastly, use of natural microbial consortia is limited to naturally occurring functions, thus expanding the use of consortia across more applications will necessitate intervention and rational designing.

Communities may instead be constructed from the bottom-up using discrete organisms combined to foster simple relationships based on, for example, division of labor, boosting of performance (i.e., some or all members of a community may individually be able to perform a function but when co-cultured, the activity of this function is greater than on the individual level), production of a secondary metabolite, or other emergent properties. Consortia built from the bottom-up, i.e., synthetic consortia, are often constructed using combinations of compatible, industrially relevant organisms (as in the above-described examples involving the industrial cellulase-producing *Trichoderma* combined with a discrete, organic acid-producing microbes). They may also be constructed by first employing a top-down approach to identify key microbes within a natural community with which to reconstruct a minimal, functional synthetic community (as in the example involving *T. asperellum* combined with *L. paracasei*)^46,47^. Though simpler, synthetic consortia are often unstable and dynamic as compared with natural consortia which have attained stability through long-term co-evolution and establishment of various chemical equilibria^7,54,55^. One study compared an enriched microbial consortia derived from the gut of a ruminant with a synthetic consortium constructed using an analogous, though much simpler, co-culture of well-characterized, anaerobic, cellulolytic fungi and methanogenic bacteria^7^. While syntrophy was successfully attained in the synthetic consortium and comparable methane production to the native consortia from lignocellulosic substrate was observed, the stability of the synthetic culture (typically losing the slower growing and oxygen-sensitive methanogenic members in less than ten passages of repeated batch cultures) was significantly outperformed by the natural consortia which remained stable for over 2 years (>200 passages)^7^. Among the possible causes of the instability, the synthetic consortium may have lacked key, co-evolved relationships present in the natural consortium including with oxygen-scrubbing bacteria and specific fungal partners with which certain methanogens can form physical associations (i.e., a biofilm)^7^. Methods for engineering stability into synthetic consortia therefore represent a key requisite for wider implementation of synthetic microbial consortia as discussed below and summarized in Table 1.

**Table 1:** A comparison of various approaches/tools for tuning microbial consortia dynamics along with exemplary references

Method	Implementation	Consortia employed	Outcomes	Advantages	Potential limitations
Quorum sensing (QS)	Linked to: cell lysis^59,60^; baceteriocin production^57,58^; growth rate enhancement^61,62,70^	Bacteria; Typically single species/multiple strain co-cultures of: *E. coli*^58,59,62,63,70^; *S. typhimurium*^60^; *L.lactis*^57^	Stable consortia with tunable compositions. Demonstrated batch processes up to 30 h^57^ and continuous processes up to 11 h^63^	Well-established and wide applicability of QS molecules	No demonstrated applications outside of bacterial systems; requires genetic engineering; “hardwired”; requires additional input/mechanism for real-time adjustment by user
Metabolite exchange	Auxotrophs engineered to exchange: amino acids^66,68,69^; TCA cycle intermediates^66^; vitamins^67^	Bacteria and yeast; Typically single species/multiple strain co-cultures of: *E. coli*^66,67^; *C. glutamicum*^68^; *S. cerevisiae*^69^	Stable consortia with tunable compositions. Demonstrated continuous processes up to 14 days^67^	Well-established and wide applicability including demonstrated applications in non-bacterial systems; doesn’t necessarily require genetic engineering	Without additional inputs/mechanisms, difficult to precisely tune consortia composition or enable real-time adjustment by user; often requires genetic engineering
Optogenetics	Linked to: antibiotic resistance^76^; antitoxin production^75^	Single species/multiple strain co-cultures of *E. coli*^75,76^	Tunable consortia, stable at least to 48 h^75^ and including a continuous process of at least 40 h^76^	Enables real-time consortia composition tuning	Limited orthogonality; not demonstrated outside of *E. coli*; requires genetic engineering; may be challenged by long-term stability
Electrogenetics	Linked to: QS^59,61^; growth rate enhancement^61^; cell lysis^59^	Single species/multiple strain co-cultures of *E. coli*^59,61^	Demonstrated to maintain stability and real-time tunability for at least 24 h^61^	Enables real-time consortia composition tuning	Not demonstrated outside of *E. coli*; requires genetic engineering; has so far only demonstrated on/off effect
Spatial niche engineering	Strategies include: use of membrane bioreactors^46,49^; compartmentalization^26,47^	Bacteria^26,46,47^, yeast^26^, filamentous fungi^46,47,49^; and plant cells^46^. Often involve cross-kingdom consortia (including natural^47,49^) comprising fungi with bacteria^26,46,47,49^ as well as microalgae with fungi^46^	Very stable consortia. Continuous production in membrane bioreactor demonstrated for at least 400 h^46^ while compartmentalization enabled reusability (10 times) and long-term storage (3 months) without loss of functionality^26^	No genetic engineering required; compatible with industrial strains; enables real-time consortia composition tuning; very stable; can enable reusability and long-term storage	May require modulation of bioreactors; cannot resolve all inter-microbial incompatibilities

Note: Included in the overview are specific strategies typically employed for a given method as well as examples of microbial species that have so far been employed for each method. Additionally, a summary of general outcomes for each method as well as a qualitative evaluation of advantages and potential limitations for each method are described. Notably, many methods below allow for, or depend on, incorporation of additional mechanisms. For example, strategies involving electrogenetics have been linked to expression of quorum sensing molecules (which in turn modulates growth rate or activates cell lysis^59,61^) as the mode of action.

To attain stable synthetic microbial consortia, interactions between consortia members may be engineered. To that end, there are two predominant strategies for tuning microbial consortia intercellular interactions, i.e., using intercellular communication mechanisms or metabolite exchange^56^. Quorum sensing (QS), for example, is a natural prokaryotic communication system wherein cells produce autoinducer molecules which regulate gene expression as a function of cell density and, in the interest of tuning co-culture population dynamics, can be integrated with pathways that, e.g., control production of bacteriocins^57,58^, induce cell lysis^59,60^, or regulate growth rates^61,62^. Organisms may be engineered to be self-regulating^58^, act on other members of the community^63^, or both (which enables, for example, gene expression as a function of strain ratio instead of overall population size)^60,63^. Moreover, effective control over population dynamics of multiple strains may be achieved using just one strain engineered for toxin production^58^. Lastly worth noting, while quorum sensing is a prokaryotic system, intercellular communication systems have long been demonstrated capable of being engineered into eukaryotic and even cross-kingdom systems^64,65^.

In addition to communication systems, metabolite exchange, which is ubiquitous in natural communities, represents another avenue for behavioral tuning^56^. For example, by making organisms co-dependent on metabolites produced by one another (i.e., syntrophic interactions), population stability is promoted^56^. Well-developed cross-feeding strategies and engineering tool kits involving auxotrophs for one or several essential metabolites (e.g., amino acids, TCA-cycle intermediates, vitamins, etc.) have been reported for bacteria (mainly *E. coli*^66,67^ but also communities involving genome-reduced *Corynebacterium glutamicum*^68^) as well as yeast^69^. One representative study involving a pair of metabolically dependent *E. coli*, additionally split a natural product biosynthetic pathway between the two strains (i.e., an upstream and downstream strain)^66^. While the metabolic co-dependence enabled population stability independent of initial inoculation ratio (addressing the much faster growth rate of one strain), the introduction of the biosynthetic pathway severely limited the growth of the downstream strain and resulted in accumulation of an intermediate compound^66^. To address this, a biological sensor for the intermediate was engineered into the downstream strain^66^. This sensor was linked to expression of a key, growth-promoting gene and ultimately resulted in significant improvement in both growth of the downstream strain as well as product titer^66^. Importantly, the outcomes of metabolic interactions between microbial community members are influenced by more than just the specific metabolites or signaling molecules being exchanged^10,70^. Entirely different outcomes can result from, for example, different medias possessing different redox conditions^10,70^. While this may seem obstructive to bioprocess design, with better understanding and further exploration, it may also represent an additional lever of control over consortium behavior^10,70^.

Many of the engineered interactions described above may be generally characterized according to one of the six fundamental biological relationships between organisms: the unidirectional relationships commensalism, neutralism, and amensalism as well as the bidirectional relationships mutualism, competition, and predation^57,71^. The stability of natural consortia is largely attributed to mutualistic or commensalistic interactions that have evolved over time while the instability of synthetic consortia is often attributed to amensalism, competition, or predation between community members (though these may also be leveraged for stability)^7,20,56^. Thus, engineering explicit relationships between members of a synthetic consortium represents an appealing strategy for achieving stability. In one study, each of the six fundamental biological relationships were engineered into six different two-strain co-culture combinations^57^. The various relationships were achieved through, for example, inducible antibiotic resistance (i.e., two organisms cultured in the presence of antibiotic where one member produces a compound that induces antibiotic resistance in the other) or production of antimicrobial compounds^57^. While the utility of engineering mutualism is clear, exploration of other fundamental biological relationships may present further opportunities for engineering tunable microbial consortia. For example, already mentioned is the general concern over losing engineered functionality (e.g., high productivity, non-native substrate conversion, etc.) over time. Thus, it would be useful if certain populations of cells known to be losing functionality could be removed and replaced with fresh cells without disruption of the overall bioprocess. The “rock-paper-scissors” methodology was demonstrated to achieve this through the programming of different populations which are each able to kill one strain and are able to be killed by another using a system of toxins and antitoxins^19^. Adding a killer strain into a culture of a susceptible strain that has begun losing function due to mutations allows for efficient removal of the old cells without disruption in the function^19^.

Also notable are efforts to engineer spatially and structurally dependent microbial interactions. In nature, the formation of specialist subpopulations is often spatially dependent, as in the case of ruminant guts, soil microbes, etc^9,72^. As mentioned earlier, spatial niches have been exploited for developing synthetic consortia comprising members with diverse biological requirements^46,47^. Hydrogel encapsulation, for example to reconcile contrasting oxygen requirements and enable co-culturing of otherwise incompatible microbes, additionally enables long term re-use and storage (e.g., via lyophilization) for on-demand chemical production^26^. The engineering of ecological interactions between microbes, as described above, also exhibits spatial consequences where organisms will colocalize or mutually exclude one another when engineered to possess mutualistic or competitive relationships, respectively^57^. This may therefore represent a useful strategy where it is desirable to control physical association between microbes. There are also synthetic tools specifically designed for programming spatial characteristics including multicellular morphologies and patterns using, for example, multiple populations of engineered cells expressing genes encoding cell-surface antigens and corresponding nanobodies, respectively^73^.

Lastly, while many of the strategies described above are effective at achieving stable and productive microbial communities, they typically do not permit simple dynamic tuning throughout the bioprocess. However, there are some recent notable examples of strategies that enable dynamic tuning including a system involving combined bioelectronic/QS control over consortium composition^59,61,62,74^. For this, an electrode is used to generate hydrogen peroxide which regulates genetic expression of signaling molecules that can in turn boost growth rate^61,62^, lyse cells^59^, or control metabolite production^74^. While this approach results in an on/off switch-like control over consortium composition (akin to, as the authors described it, a thermostat^47^), other strategies can enable control over a range of outputs. For example, the systems involving spatial niche engineering can, as mentioned, exploit concentration gradients (e.g., of oxygen) which form across niches^46^. Since these concentration gradients are a function of the gas feed and since they dictate bio-spatial characteristics within the reactor, they present a unique opportunity to precisely tune bioprocess parameters in real-time (e.g., through adjusting flow rate of the feed). Additionally, optogenetic regulation represents a relatively recent and increasingly reported approach which enables dynamic control over population composition by linking blue light input with expression of, for example, a toxin/antitoxin system^75^ or an antibiotic resistance-conferring enzyme^76^. Optogenetic control may easily be integrated into a real-time closed loop feedback control wherein the light input is linked to measurements of the state of the cells in the culture.

## Strategies and tools for modeling and measuring microbial communities

Common in the above-described studies is the development of computational models to describe microbial community dynamics as well as predict dynamics under different conditions or with increasingly diverse composition^20,57,59,60,62,63^. Experimental data from relatively simple two- and three-strain communities may be effectively employed to predict the population dynamics of up to seven strain cultures^57,77^. Entirely in-silico models have also been proposed to predict optimal two strain and three strain co-culture designs based on a variety of QS/bacteriocin interaction mechanisms^20^. Notably, for both two- and three-strain communities, the top-performing models involved mutualism and self-limiting interactions; for example, where a subpopulation produced a bacteriocin that was harmful to itself but could be alleviated by interaction with another subpopulation^20^. In the future, bottom-up model designing as described above may benefit from more advanced top-down models currently driven in large part by a growing awareness of the importance of wild microbial communities in environmental processes (e.g., biogeochemical processes) as well as human health (e.g., human gut microbiome). These models mainly aim to explain diversity and stability in natural communities^78^, and may ultimately provide insight which enables construction of more complex, highly diverse synthetic communities. While predicting microbial interactions is key to designing stable consortia, other models aim to maximize microbial functionality (e.g., increase product titers, yields, and productivity or microbial growth rate). These may be constructed based on stoichiometric/metabolic principles and benefit from the vast amount of metabolic reaction data available as a result of the past few decades of work in whole-genome sequencing^10,79^. Metabolic models are developed to identify key bottlenecks in metabolic pathways and inform engineering strategies for maximizing carbon/energy/electron flux toward the desired function, for example, production of fatty alcohols ultimately from lignocellulosic substrate^79,80^. Stable isotope tracing techniques are widely employed to investigate metabolic network models, i.e., ^13^C-metabolic flux analysis, and have demonstrated application in multi-strain, microbial biofilms^81^. These techniques aid in maximizing carbon and energy flux toward some desired functionality, but what is also urgently needed for facilitating the use of synthetic microbial consortia in commercial applications are models for optimizing bioprocesses, e.g., to find maximally productive conditions for multiple strains that have contradicting optimal environments^82^.

Better models and continued development of tools for tuning microbial consortia behavior and productivity depend on a continued improvement in understanding consortia dynamics, which, in turn, requires new strategies for extracting information from microbial communities. For example, spatial heterogeneity is one exploitable feature of microbial consortia, but spatially dependent interactions are complex and difficult to analyze. Microfluidic devices, fabricated to facilitate spatial analysis of microbial consortia, have recently presented utility in this endeavor. One such reported device enabled spatial segregation of two populations of cells (though modification of the design could enable more) between which molecules could diffuse and permit investigation of the effects of distance on stability, functional activities, and responses to environmental perturbations^72^. Microfluidic-based techniques have also been applied in continuous microbial communities (i.e., biofilms). For example, subpopulations of cells within a microfluidic device-cultivated biofilm were successfully sorted according to location in the biofilm and separately analyzed^83^. Through a combination of live staining and machine learning-guided cell sorting, it was determined, among other things, that the nutrient poor biofilm interior was, contrary to prior belief, still active^83^. The live staining method employed in this study was dependent on the specific biology of bacteria^84^ (i.e., incorporation of fluorescently labeled amino acids into peptidoglycan of cell wall) and thus development of similar techniques with applications to other kingdoms of life (e.g., fungal and plant cells) would be valuable.

There is additionally a need for improved methods of real time monitoring of process parameters (e.g., population composition, substrate/product/inhibitor compositions, dissolved gases, etc.) within microbial consortia. This would both enable optimal implementation of the expanding synthetic toolbox for tuning microbial consortia behavior described earlier, as well as improve commercial/industrial attractiveness of consortia-based processes. Microbial consortia-based bioprocesses present unique challenges (e.g., the need to distinguish species), and moreover, these challenges are exacerbated by complexities inherent in lignocellulose conversion (e.g., the presence of solid particles)^85^. A variety of techniques are available for elucidating population composition^54^ and measuring chemical/biochemical parameters in lignocellulosic processes^85^, but few are implementable at-line and fewer online with the bioprocess. Optical techniques including light scattering are among the few which present high potential for online implementation and have demonstrated utility in non-invasive co-culture population monitoring^86^. Raman spectroscopy, which is based specifically on the inelastic scattering of photons, is widely used for non-invasive monitoring of commercial cellular processes^87^. While there are no reports of its use in characterizing/monitoring microbial consortia, it has been shown capable of distinguishing between different phenotypes (i.e., stressed and non-stressed) of the same microbial species and thus demonstrates potential for use in distinguishing different members of a consortium^88^. However, such optical methods are challenged where lignocellulose is used as substrate since typically the presence of solid particles will interfere with spectroscopic and image-based analyses^52^. Methods involving fluorescently tagged organisms have demonstrated utility in resolving population dynamics in lignocellulosic based processes including processes involving filamentous organisms which present additional complications to monitoring population dynamics^89^. Where optical techniques cannot be employed, there are other approaches to measuring bioprocess parameters in real time including off-gas analysis via mass spectrometry. This approach has been employed in, for example, measuring substrate composition/product formation as a convenient method for optimizing substrate feeding^52^.

**Fig. 5:**
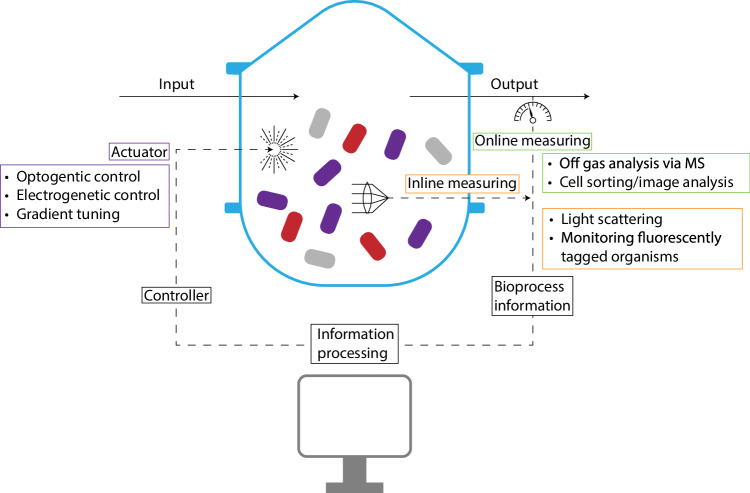
Control loop for microbial consortia-based bioprocess. An essential control loop comprises a means of extracting information from an ongoing process, processing this information, and adjusting parameters accordingly in real-time to attain a desired outcome. Recent work has demonstrated methods for effective online (and at-line) measuring of various parameters in microbial consortia bioprocesses, including those involving lignocellulosic substrate. These measurements may be made at the outlet or within the reactor itself. Furthermore, optogenetics, electrogenetics, and spatial engineering (i.e., with use of abiotic gradients) represent actuators that may be employed to adjust parameters in real-time in direct response to measurements.

Ultimately consortia-based bioprocesses must feature a control loop wherein population behavior is monitored and then control is exerted over that behavior when needed, as outlined in Fig. 5. What this might look like in practice has recently been demonstrated using a system of optogenetic feedback^76^. This study describes a method for co-culturing two naturally unstable strains of *E. coli*, the faster growing of which was engineered to differentially express an antibiotic resistance-conferring enzyme when stimulated with light, referred to as the photophilic strain^76^. Samples are periodically and automatically taken from the culture and analyzed for population composition via cell sorting which in turn modulates the light intensity to directly control the growth rate of the photophilic strain^76^. A number of challenges will need to be addressed for wider application, including the loss of control after 40 hours (likely due to mutation), requirement for orthogonal optogenetic tools to use in more complex cultures^76^, and problems which may arise where a complex substrate like lignocellulose is desired. However, this system represents an exciting early example of how co-culture control loops may be constructed. Other tools that have been used for tuning consortia dynamics present future opportunities for integration into a control loop including, as mentioned, electrogenetic^61^ and membrane-based niche systems^46^ in which electrode generated signals and concentration gradients (including of gases, nutrients, light and/or other abiotic factors) respectively have the potential for real-time adjustment.

## Outlook and conclusion

The availability of biomass, though cheap and relatively abundant, has historically been vastly overestimated and thus lignocellulose, at least technically and sustainably available lignocellulose, should be considered a scarce resource^90,91^. Additional defossilized carbon feedstock streams, e.g., CO_2_, may therefore be necessary to supplement the input of lignocellulosic biomass. Biological methods for harnessing CO_2_ mainly comprise photosynthetic conversion of CO_2_ into, for example, lipids by algae and cyanobacteria. This process of biological conversion of CO_2_ into products is slow although organic carbon substrate can be supplemented to elicit mixotrophy and improve growth rates^92^. Typically, relatively expensive sources of organic carbon are employed for this purpose and thus adopting cheaper lignocellulosic substrate may serve to improve process economics. However, while some photosynthetic organisms have been shown to produce small amounts of cellulolytic enzymes^93^, they do not catabolize/convert lignocellulosic biopolymers sufficiently enough for application. Fungi of course are well equipped for catabolizing lignocellulosic biomass and are also known to form synergistic relationships with photosynthetic organisms in nature, i.e., lichens. Engineering synthetic lichen may therefore enable simultaneous, effective utilization of lignocellulose and CO_2_, though this has been reported to a very limited extent^46^. More generally, co-culturing of a fungus (*Aspergillus nidulans*) with a polysaccharide-secreting cyanobacterium (*Nostoc*) has been shown to boost lipid production in the photosynthetic partner^94^. Co-culture systems have also been developed wherein the photobiont, *Synechococcus elongatus*, is engineered to export sucrose or, more recently, glycerol which then serves as a carbon source for a production microbe to generate, e.g., 3-hydroxypropionic acid^95^, lactate, 1,3-propanediol, and polyhydroxybutyrate among others^96^. Another option for photosynthetic co-cultures, may be to substitute the biological with an abiotic photosynthetic component. For example, one study reports the use of a photovoltaic electrolyzer to generate syngas from CO_2_ and water which was then converted via anaerobic co-culture (*Clostridium autoethanogenum* and *Clostridium kluyveri*) into butanol and hexanol^97^. The authors propose that given the need for a high CO_2_ feed concentration (i.e., higher than atmospheric), such a setup would ideally be decentralized and, moreover, combined with the exhaust CO_2_ from, for example, breweries and biomass conversion facilities^97^.

The features which characterize microbial consortia (i.e., specialization of tasks and distribution of molecular burden) make them well-suited to the complex task of lignocellulosic biomass conversion. Moreover, much as it seems the energy sector will require a multi-form approach to meeting the world’s renewable energy needs (i.e., with wind, solar, hydro, etc.), so too may it be necessary to have multiple carbon streams to produce all the chemicals demanded globally. To that end as well, microbial consortia represent a potentially excellent strategy to unlock the carbon available in CO_2_ in addition to lignocellulose. While natural microbial communities exist which may be exploited to convert biomass into, for example, biogas, synthetic consortia will be needed to expand applicability. However, these communities can be complex and unstable, thus commercial adoption requires development of effective tools for monitoring and control. Many recently developed synthetic tools enable effective stabilization and tuning of microbial consortia but have only been applied in bioproduction to a limited extent, much less in conversion of lignocellulosic biomass and CO_2_. Once robust mechanisms for controlling microbial consortia are available, and once outstanding bottlenecks hampering lignocellulosic conversion in general (e.g., conversion of all feedstock components into product, consolidation of processing steps, effective means of real-time process parameter monitoring, etc.) are alleviated, wider commercial adoption may not be far off.
